# The qualitative TOL: integrating trees, networks, and organismal biology

**DOI:** 10.1007/s40656-026-00771-0

**Published:** 2026-11-10

**Authors:** Amadeo Estrada

**Affiliations:** Facultad de Ciencias (Sciences Faculty), Universidad Nacional Autónoma de México (National Autonomous University of Mexico), Mexico City, Mexico

**Keywords:** Tree of life (TOL), Network of life (NOL), Last common ancestor (LCA), Lateral gene transfer (LGT)

## Abstract

The tree of life (TOL) was, for almost 150 years, conceived as the representation of the history of life. Since the discovery of lateral gene transfer (LGT) and its relevant evolutionary implications, the TOL has been questioned as a correct representation and several researchers have proposed networks of life as a suitable replacement. Nevertheless, these depictions pose important epistemological problems, such as historicity and resolutions levels. This paper problematizes on such subject and sustains that both representations, the TOL and networks, necessarily collide and correspond to different types of inheritance that require distinct levels of resolution. I argue in favor of doing such a differentiation for understanding and modeling diverse biological processes. I analyze here different representations of the history of life, their meanings, and several inherent problems to some of them. Substituting the TOL by networks of life as the correct representations, or sustaining the TOL notwithstanding the amount of LGT evidence, obliterates important data. This paper proposes a *qualitative* TOL, a TOL produced by core and essential genes and organismic information, that serves as a necessary scaffolding for anchoring LGT networks and pivotal for providing a historical dimension in our representations. An important part of the paper is the historical parameter of the different representations. An argument about why networks without the TOL fail to retrieve historicity is made. Finally, the *qualitative* TOL serves as a tool that gives orientation through the entangled data in order to understand the webbed history of life.

## Introduction

Reconstruction and representations of life history affront several challenges. Todays’ knowledge of genetics has made us rethink about the best models to understand and represent the biological evolutionary history. Having discovered the effects of Lateral Gene Transfer –LGT– in genes distribution has opened a major discussion in the field about representations, from sustaining the old Darwinian idea of the Tree of Life –TOL– to proposing a new and, for many, more correct graph depicting networks, the result of LGT between clades. In this paper I argue about the possibility of assembling both together, and the different resolutions that each of these graphs generates. The substitution of one model for the other may be appealing but it also produces the loss of important information. I defend that lineage chronology is retrieved by the data that produces a TOL, and that networks cannot deliver such a result. I also discuss about the genetic-statistical stand in Biology, which has given, along with relevant results and the possibilities to explore data previously inaccessible, also the impression that it is the most accurate and sufficient source of information. I propose to complement with organismal Biology in order to better understand evolutionary processes and propose a *qualitative* TOL as productive for these endeavors.

In this paper, I argue in favor of the conjunction of organismal and genetic data as a tool for better understanding the evolutionary history of life. I introduce the *qualitative* TOL as an integrative model produced by genetic information and cellular, lineages data; this idea is carefully explained in Sect. 5. My point is focused on the representation of historicity, which I think is not accurately achieved by a genetic-statistical approach, with data of genes dispersion. For this, the debate that has been occurring in Biology since the late 1990s between different stands in the matter of representations of the history of life is presented. There are proponents of networks of life, as well as those that defend the TOL.

First, in Sect. 2, *Historical antecedents*, I bring a brief overview of the available representations. Then, in subsect. 2.1, *LGT and the representations of the history of life*, I present some relevant differences between the organismic perspective and that of the genetic-statistical approach, and exhibit that the use of different kinds of data has resulted in different representations of contrasting processes and meanings. In Sect. 3, *Current types of representations*, various representations of the TOL and of networks by several authors are shown. In Sect. 4, I discuss how a quantitative approach does not represent the qualitative characteristics that belong to the TOL, nor to networks. The main subject in this section is about the different biological processes and different levels of resolution, meaning that data from genetics and genomics—sequence comparisons—have a different resolution than data from organismal Biology (subsect. 4.1). Networks and the TOL refer to different types of inheritance and of evolutionary processes, they should not be conflated. In this sense, I refer to levels of resolution as the representation of quantities of genes, entire genomes versus small sequences, and the types of inheritances that produce them: vertical inheritance—produced by reproduction, asexual and sexual—or lateral gene transfer (LGT). Do we have to choose between the network representations and a TOL, or can we assemble them together? If so, how? (subsect. 4.2).

In subsect. 4.3, I discuss why, without the TOL, networks are unable to give us a historical order (historical polarity). There is a relevant qualitative, organismal, dimension into this discussion. With this I defend that if we want to make the case for networks as a representation of the history of life, and therefore represent an important biological process, that of LGT, we are obliged to imbricate them into the TOL. Without the TOL as a scaffold, networks of LGT become an elusive and an ahistorical representation, because the only means we have to establish historicity is by tracing vertical heredity. Finally, in Sect. 5, I propose a *qualitative* TOL, coherent with organismal Biology, and genomics, and also as the necessary scaffold for genetic networks. Such a model integrates different levels of resolution, from biological clades, concordant with organismic Biology, to a genetical one that integrates specific genes laterally transferred. I discuss why this model can have advantages over a genetical-statistical approach, as is O’Malley and Koonin’s (2011) proposal of the *statistical* TOL. I argue that organismic history is not adequately retrieved by a merely genetic-statistics. In this sense, my proposal of a *qualitative* TOL differs from other previous models. The genetic-statistical approach is unable to produce the desired results for representing the history of life without organismal data as a counterbalance. Biology is often more than just statistics.

## Historical antecedents

Darwin’s idea of the TOL was an attempt to represent the reconstruction of the history of life, given that a tree structure is vastly concordant with vertical inheritance and branching patterns of natural selection and other evolutionary processes (Darwin, 1859). However, this idea lacked substantial empirical data during Darwin’s time, and remained so until a century later. The Darwinian idea of a TOL, until the 1960s and 1970s, came from the perspective of organismal Biology. Nevertheless, the molecular and genetic Biology allowed the possibility to search into such a concept with empirical data from all life main groups. In the 1960s, Zuckerkandl and Pauling published a series of papers that strongly influenced Biology, and convinced practitioners in the field that genetic markers not only made it possible to tackle such questions, but also that they were capable of reconstructing the history of life (Zuckerkandl & Pauling, 1965; Doolittle, 1999a; Suárez–Díaz, 2017, Zuckerkandl, 1964—in Suárez–Díaz, 2017). Consequently, the proximity among life groups could be established, giving the idea of a TOL a more refined and testable approach for the very first time. These ideas were seminal for further attempts at producing a genetic TOL that would represent, better than any organismal data, the TOL that Darwin referred to, substituting any previous representation. Such data provided what no other discipline in Biology had ever supplied for representing the history of lineages.^1^

In the 1970s, with Woese and Fox’s paper (1977), the reconstruction of the TOL became foreseeable. This research took one of the most conserved traits in life, the small subunit rRNA sequences, and compared it among different biological groups. The result of such comparisons consistently yielded three-groups partitions which we call today the three Domains, an idea much in congruence with the TOL. It was due to this research that biologists began to work on the model of the three major groupings of life, where a third group, Archaea, was proposed (Woese & Fox, 1977 and Woese et al., 1990). Furthermore, Woese and Fox’s work also became the first research endeavor that allowed an approach to the idea of a common ancestor for all extant biodiversity (Woese & Fox, 1977) from empirical data. They called such ancestor the *progenote*, a concept that has changed into different ones with further characterizations and which is broadly called today the Last Common Ancestor (LCA).^2^ Although the LCA differs from the *progenote*, both pursue the Darwinian proposal of a common ancestor for all today’s biodiversity. After almost 120 years since Darwin’s proposal, such research subjects had remained unreachable from an organismal perspective, but the powerful usefulness of molecular and genetic sequence comparisons became incontestable for these purposes. Whereas the TOL and a common ancestor for all extant biodiversity are concepts produced by organismal Biology, they did not become accessible until molecular and genetic sequence comparisons were developed. This represented an empirical replacement of the tools –from organismal to molecular Biology and genetics– to attempt such concepts, which implied also different experimental subjects.

### LGT and the representations of the history of life

Since the late 1990s, also from sequence comparison practices—specially from studying phylogenies–, several research programs have sustained that the history of life might be better represented by networks, rather than by the TOL (Doolittle 1999a, b, 2000; Bapteste et al., 2009; Bapteste & Dupré, 2013; Bapteste & Huneman, 2018). These studies have assessed the presence of weblike links between genes of well differentiated clades, which in fact result in an intricate web inside each Domain and between all three of them—Bacteria, Archaea and Eukarya–. The basis for such webs lies on the effects produced by LGT. As early as 1999, Ford Doolittle pointed out the outcomes and implications of LGT, if it was found to be a major phenomenon (Doolittle, 1999a). Our current knowledge has shown that it is indeed an important biological process with relevant implications (Dagan & Martin, 2006; McInerney et al., 2008; Sapp, 2009; Bapteste et al., 2009; O’Malley, 2010, 2018; O’Malley et al., 2010; Bapteste & Dupré, 2013; Bapteste & Huneman, 2018). One germane result is the formation of webbed phylogenies when using genomes, varied specific groups of genes or molecular data. Accordingly, and since more than twenty years ago, Doolittle has called for a change in evolutionary representations, by transitioning from a TOL to network models, since these would be a more accurate representation of the history of life. An important fact to be considered, however, is that some single universal genes point towards tree structure phylogenies, not to networks. Nevertheless, several-gene and genomes comparisons result in network structures, rather than trees. Phylogenies show widely spread gene dispersions amongst the three Domains, from which biologists infer that such genes have passed from one biological group to another through LGT. This produces new challenges: where did a certain gene originate? Can we clearly discern between donors and recipients? Can we disentangle such a web?

It is worth noting that the TOL is a strictly bifurcating model that has a clear direction (time), growing from a wide trunk to fine branches. Networks, on the other hand, are reticulated and do not necessarily have the above-mentioned properties of the TOL. Nevertheless, since the first proposals of new networks of life representations, several authors have presented them as a conjunction of bifurcations—as the TOL–, and reticulations—as networks are–. This is discussed in this paper with due clarifications, in Sect.  3—*Current types of representations–*, where I describe the main types of representations that have been proposed. All of them are, in fact, networks, since representing LGT, with lines that connect branches, already transforms the graph into a network. However, some authors argue in favor of a discernible TOL as the principal structure of such a net (for example, O’Malley & Koonin, 2011), while some others propose the contrary: that any reminiscence of the TOL disappears into the networks of life.

## Current types of representations

Before proposing any representation, it is relevant to review the main and latest depictions that imply or defend networks. Representations also express—at times clearer than words—what we understand of theoretical problems or of certain phenomena. Ford Doolittle’s well-known characterization of a network of life (Doolittle, 1999a) has been influential in further network-like representations and is also an inspiring image to discuss upon.

There have been different takes on the TOL vs. networks discussion. Today many accept a certain TOL coexisting with networks. For some, networks are the basic structure upon which a scarce TOL can be attached to or form part of (Doolittle & Bapteste, 2007; Dagan & Martin, 2006; Bapteste et al., 2009; Bapteste & Dupré, 2013; Bapteste & Huneman, 2018). For others, there is a basic TOL to which scarce or profuse networks can be attached. The latter position is in favor of the possibility of reconstructing the TOL (Jain et al., 1999; Nesbø et al., 2001; Puigbò et al., 2013), as well as, therefore, the LCA (Becerra et al., 2007; Weiss et al., 2016; Crapitto et al., 2022, among others). Reconstructing the LCA is a consequence of the TOL model, for which there are possibilities of achieving some *slim* reconstructions (Estrada et al., 2022). In a network representation, reconstructing the LCA faces unsurmountable problems, and can hardly be justified.^3^ However, the idea of different webs for representing several biological phenomena is appealing for many different biological subjects, a well described matter (Bapteste & Huneman, 2018).

In my assessment, the differences between the idea of a TOL and networks arise mostly because of two main aspects: (1) Since Darwin, the TOL has been characterized by bifurcating branches in the vertical direction. The base of the TOL corresponds to the common origin of biodiversity, rising up until todays’ species, at the tree crown. The TOL, thus, implies chronology.^4^ LGT, on the other hand, produces a reticular pattern between different TOL branches. (2) In the TOL, the base –trunk– is the widest part of the tree and grows upwards while bifurcating into thinner branches. Since the early, naturalistic, representations of the TOL, major branches indicate higher clades and thinnest branches symbolize the smallest taxonomic divisions, as species. If we translate this idea into genetic representations, genes transferred laterally are not equivalent, in *weight*, to the whole genomes of a species.

If we want to add the historical transference of genes into representations, LGT lines should correspond in weight to the number of genes transferred, very few at one point in comparison to vertical heredity (the main structure of the TOL), which produces the transference of whole genomes. Therefore, there should be a more appropriate representation of branches and branching because of the number of genes pertaining to genomes. This is concordant with the representation of a single clade diverging. Some of whom defend networks over the TOL, depict in their graphics the same weight in lateral lines (LGT) than in vertical ones—vertical inheritance– (TOL). This is biologically unsupported.

**Fig. 1:**
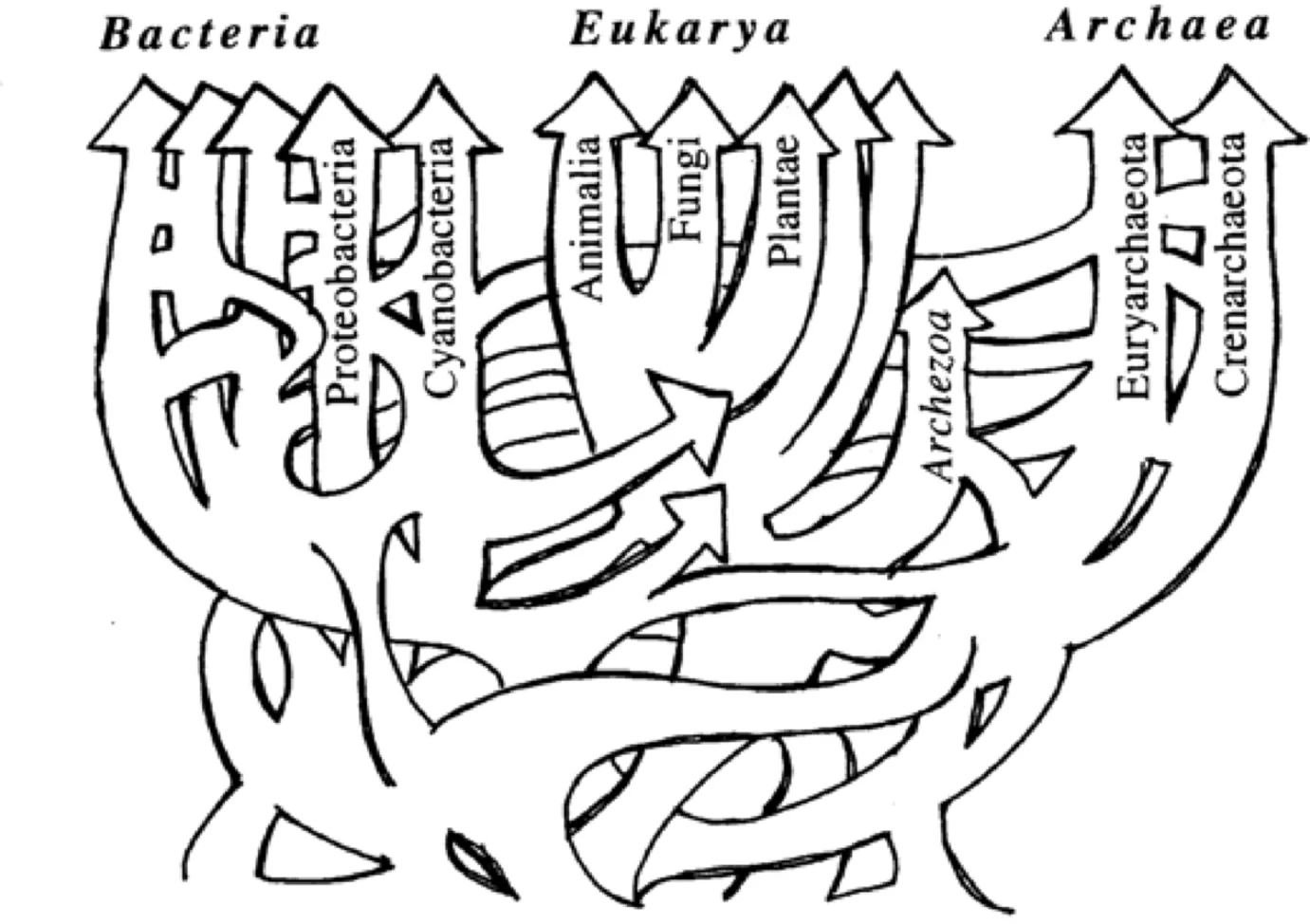
“A reticulated tree, or net” (Doolittle 1999a). Reprinted from American Association for the Advancement of Science, Vol 284/issue 5423, Ford Doolittle, Phylogenetic classification and the universal tree. /Page 2127, Copyright (1999), with permission from American Association for the Advancement of Science

**Fig. 2:**
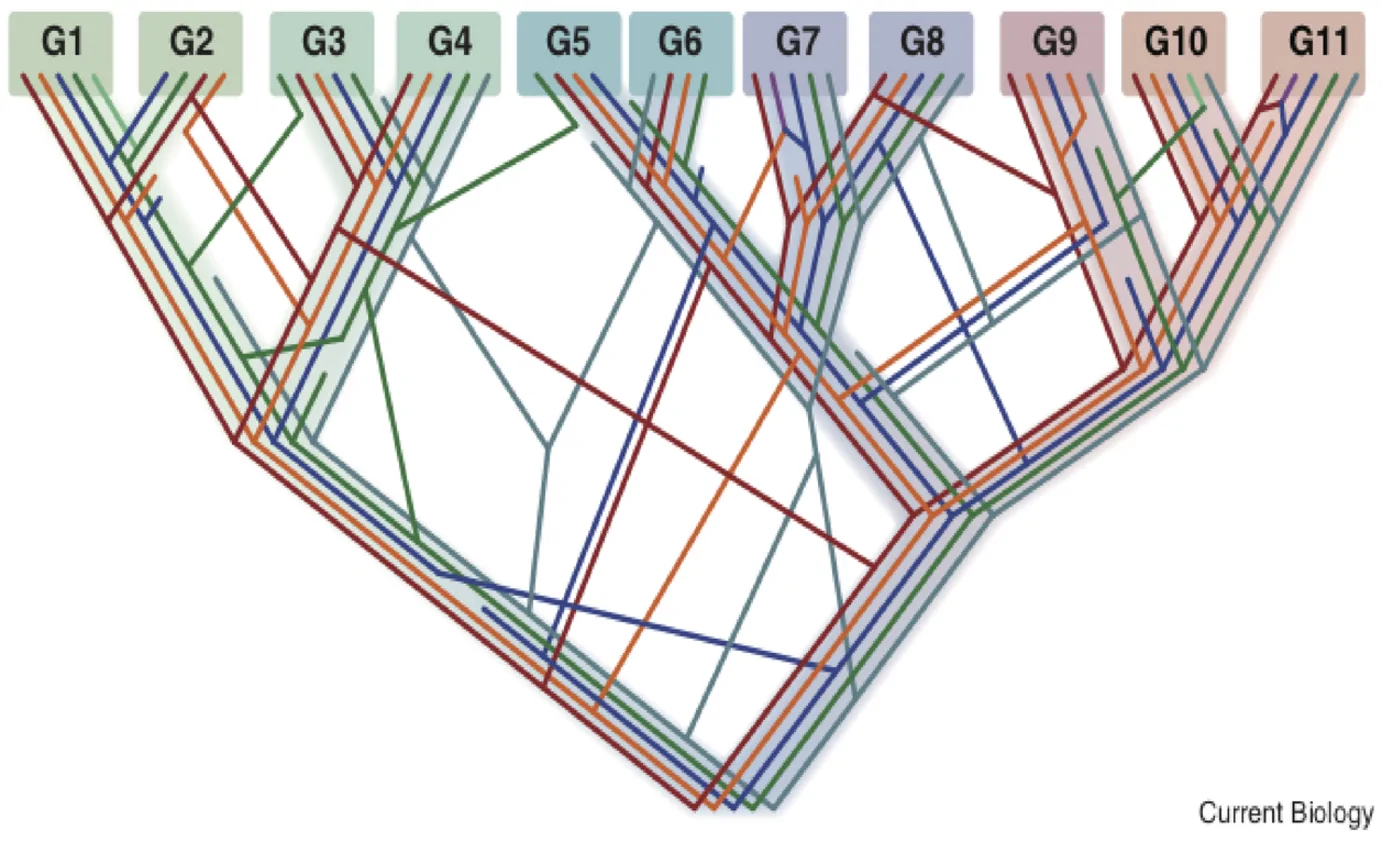
“The statistical tree of life” (Blais & Archibald, 2021), a case of a *diagonally webbed tree*. Reprinted from Current Biology, Vol 31/issue 7, Blais, Cédric; Archibald, John M., The past, present and future of the tree of life/Page 316, Copyright (2021), with permission from Elsevier

**Fig. 3:**
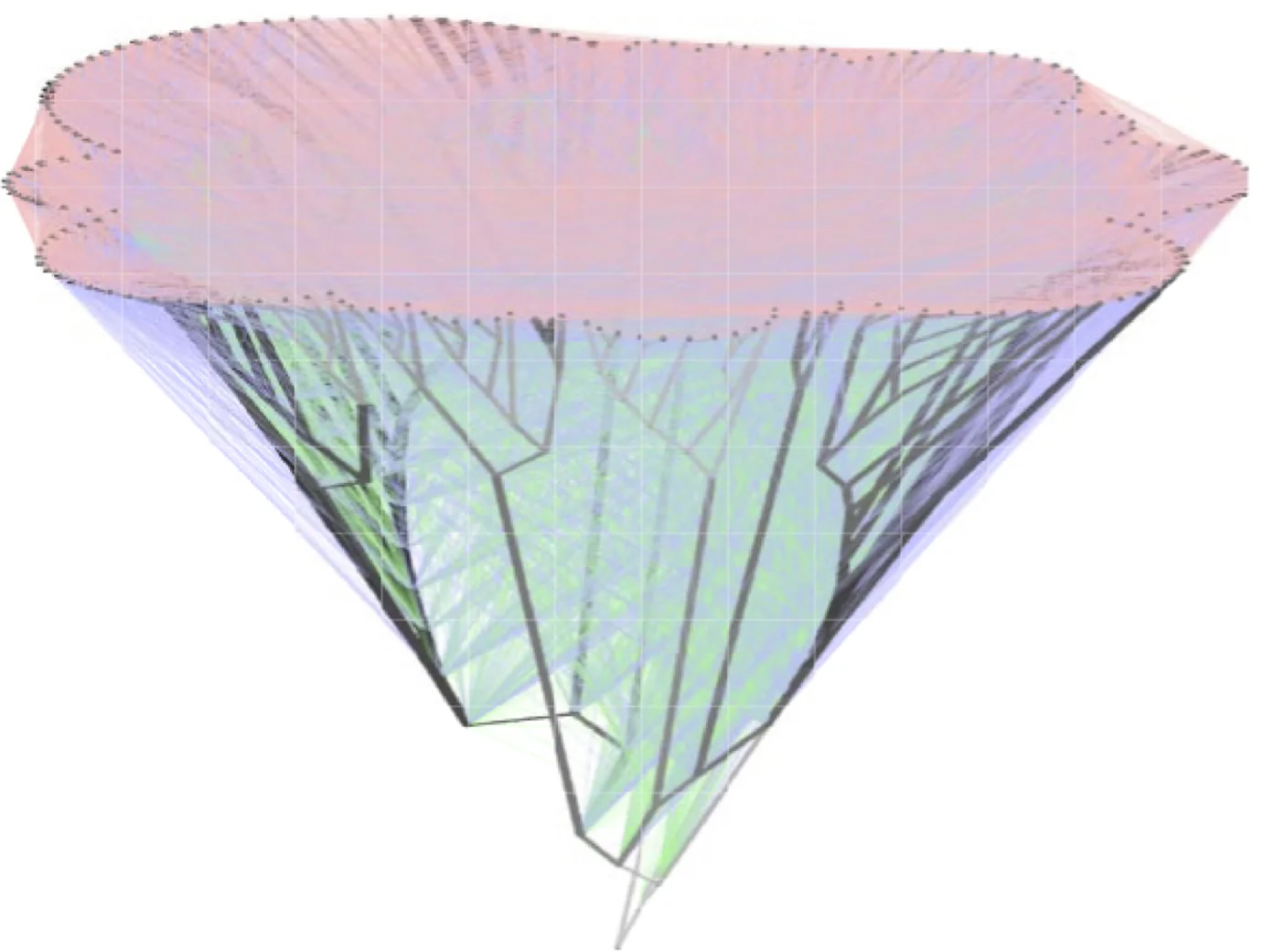
“A three-dimensional projection of the MLN”, minimal lateral network, (Dagan et al., 2008)

**Fig. 4:**
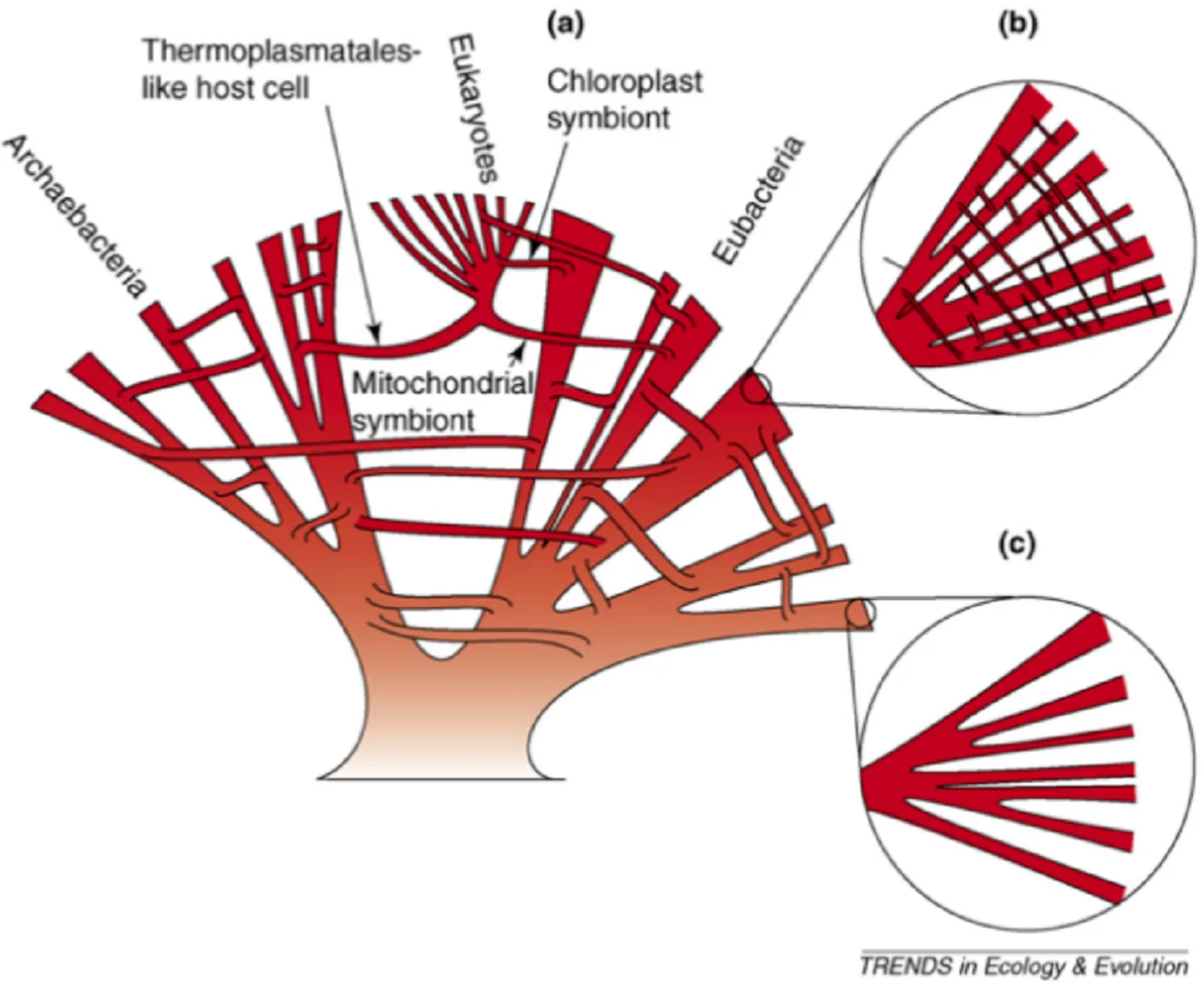
“A network of life” (McInerney et al., 2008). Reprinted from Trends in Ecology and Evolution, Vol 23/issue 5, McInerney, James O., Cotton, James A., and Pisani, Davide, The prokaryotic tree of life: past, present… and future? /Page 278, Copyright (2008), with permission from Elsevier

**Fig. 5:**
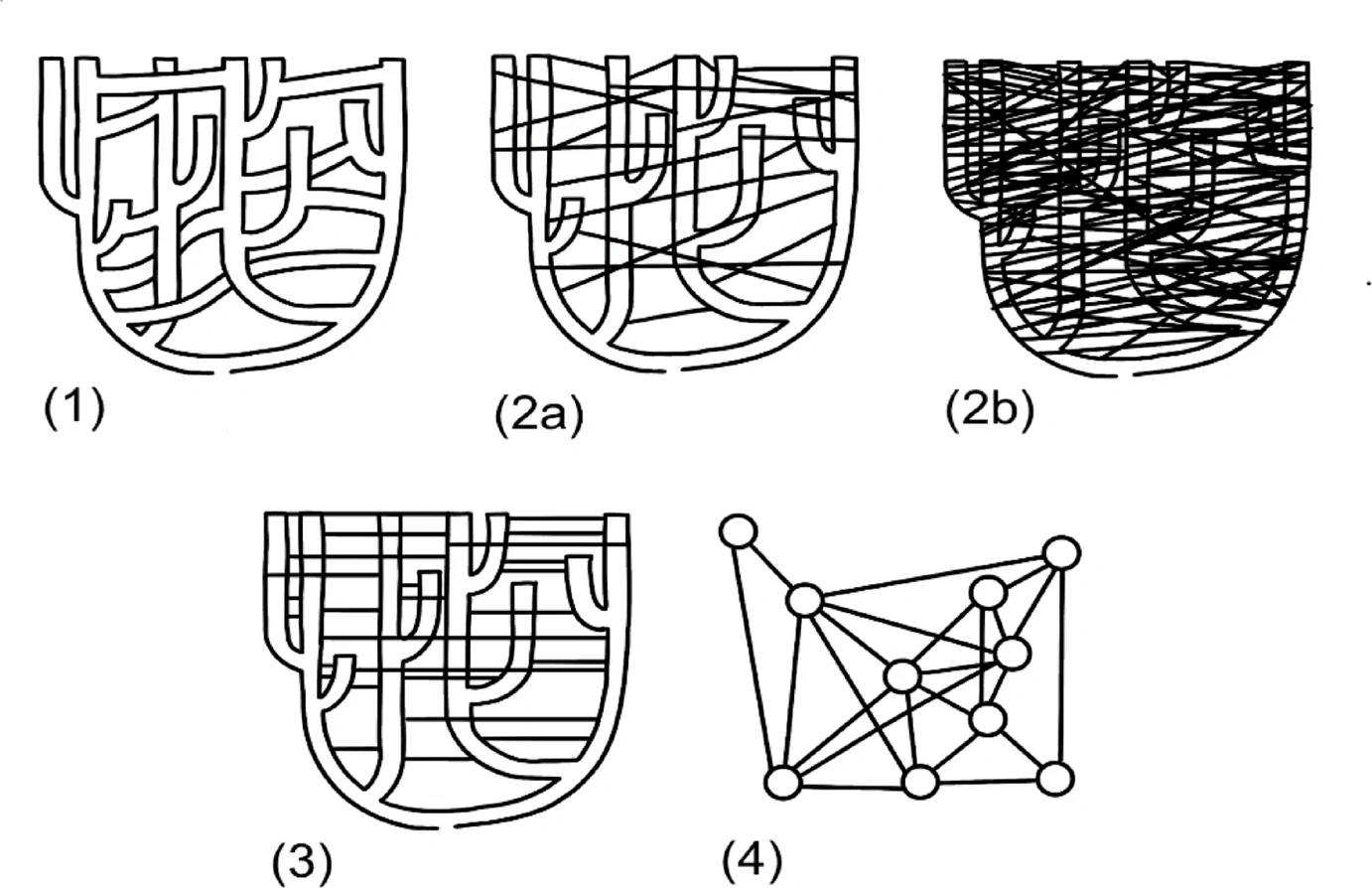
Shown here are simplified versions of some of the main types of representations found in the literature on the effects of LGT in the TOL or Webs models. (1) represents the *Noodle webs*; (2a) and (2b) are *diagonally webbed trees*; (3) represents the *horizontally webbed TOL*; and (4) represents a network of genome nodes. The original representations are richer in details and often also in implications

1) There are representations in which network lines are as wide, *heavy*, as those of the TOL, while exhibiting many ascending diagonal lines that produce a network—reticulation—of colliding branches (Figs. 1, and 5, case 1). Doolittle’s (1999a) and, (2000) representations, as well as Papale’s et al. (2020) would fit this description. I will use the term *noodled web* for such representations, with wide branches from LGT portrayed as massive as hybridization between entire groups—as high as Domains, and as wide as those of vertical inheritance–, in which ascending diagonal branches connect with other branches. These representations suggest these phenomena would have been common in life history. It must be highlighted that this is a suggestive image that has been paramount for introducing the discussion on networks versus the TOL.

2) Other representations opt for taking the TOL—strictly bifurcating graph—as a base and uniting the TOL’s branches with fine LGT lines that produce reticulation. These include the presence of ascending diagonal fine LGT lines. In this type of representation, we find two major differences: those that opt to present a scarce trace of LGT (Figs. 2, and 5, case 2a); this is the case of Smets and Barkay’s (2005), Kunin’s et al. (2005), Puigbò’s et al. (2013) and Blais and Archibald’s (2021) used it for explaining the main idea of the *statistical tree of life*, Fig. 2. The other cases are those that opt to represent a profuse amount of LGT (Fig. 5, case 2b), although with finer lines. This is quite different from the *noodled web*, like Dagan’s et al., (2008)—also referred in Swithers et al., (2009)–, as well as Swithers & Katz, (2013). Both, scarce or profuse LGT models, fit the description of a *diagonally webbed tree *(Figs. 2 and 3).

3) Another take on this problem is that of authors like McInerney’s et al. (2008), Yue et al. (2012)—although the central interest in their paper is on plants, they do provide a general TOL representation–, and Soucy et al. (2015). These all have in common a basic TOL with scarce, but significantly, horizontal–only lines, representing LGT as the union of wider TOL branches, in a *horizontally webbed TOL* (Figs. 4 and 5, case 3).

4) Finally, Halary et al. 2010 (in Blais & Archibald, 2021) describe a network of genomes nodes. This represents dispersion of genes, specifically, although it does not represent the TOL. This is, possibly the best representation of only networks data and it is strictly reticulating (Fig. 5, case 4). I will not center any further in this reconstruction, as I am discussing the TOL problem. In Fig. 5, I summarize the main different kinds of representations from previous works, under a standardized type of representation. In the next sections, I will problematize some of the theoretical implications inherent to most of these different types of representations, with the terms I have assigned to each of them in the previous descriptions.

### The genetic-statistical approach used as the criteria for choosing a TOL or networks

The discussion between supporters of a TOL and those of networks is based on a genetic-statistics problem—sequences comparisons data–, and their difference lies on the amount of LGT some think represents the problem best. Some research groups defend that LGT might not be sufficient to blur or render the TOL unrecognizable. Papers by Kurland’s et al. (2003), as well as by Puigbò’s et al. (2013) are helpful for exemplifying this posture. The main arguments for this position are based on downplaying the quantitative aspect of LGT and therefore its importance, considering it a phenomenon that might be less common than what networks proponents assume. From the same quantitative stand, defenders of networks have argued and described LGT as a profuse biological phenomenon with extensive results in biodiversity (Dagan & Martin, 2006; Doolittle & Bapteste, 2007; Bapteste et al., 2009; O’Malley, 2010, 2018; O’Malley et al., 2010; Bapteste & Dupré, 2013; Bapteste & Huneman, 2018). Nevertheless, quantification of LGT is problematic in and of itself, because of two major challenges: one is the vastness of data, with the computational difficulties attached for its evaluation, although recent technology provides much better resources. The second, and more important, is the algorithms sensibility to recognize the similarities, as a laterally transferred gene can evolve differently in the recipient group than in the donor group (Cortez et al. 2005 provides information about the troubles for evaluating LGT). However, some attempts have been made—and attest to such difficulties–, based on phylogenetic reconstructions as the main source of information (McInerney et al., 2008; Gogarten et al., 2009). Although there is a lack of precise evaluation, the data points to a recurrent phenomenon in the history of life, that has resulted from a large variety of processes (Gogarten et al., 2008). Some important precedents that contribute to this discussion around the TOL vs. networks hypotheses have been retrieved and argued in Blais & Archibald, 2021.

Such a quantitative perspective, dominant in the area, belongs to what Hagen problematized upon and called the *statistical frame of mind* (Hagen, 2003). This stand has permeated and quite frequently become the center of the TOL vs. networks discussion, as for example, in the famous 1% argument sustained by Dagan and Martin’s (2006) paper:

Fair enough, one might say, what is there to debate? Lots. Bearing in mind that an average prokaryotic proteome represents about 3000 protein–coding genes, the 31–protein tree of life represents only about 1% of an average prokaryotic proteome and only 0.1% of a large eukaryotic proteome. Thus, the positivists can say that there is a tree of life after all: a bit skimpier than expected, but a tree nonetheless. But the microbialists, glaring at the same data, can say that the glass is only 1% full at best, and more than 99% empty! There might be a tree there, but it is not the tree of life, it is the ‘tree of 1% of life’. (Dagan & Martin, 2006)

My critique to such approach centers in two points: (1) How is that 1% calculated? Our calculations of LGT are questionable. And (2) Giving all the relevance to the numeric aspect, percentages of genes, cannot grasp the history of lineages, a problem discussed in Sects.  4 and 5.

When representing the history of life only with genetic-statistics, several groups will be misidentified, and important organismal aspects will be unattended. Traits such as the cell membrane, the transcription and translation apparatuses, glycolysis, glutamic acid, lipids, pyrimidines and purines catabolism and anabolism (Rivas et al., 2018), are consilient with Woese’s three Domains, with a TOL. These are some of the basic-to-life characters that a “1%” argument aspires to erase in defending an only-numeric standpoint. Such qualitative subjects are not negligible, for these genetic-quantitative perspective lacks the needed resolution. The “99%” of shared genes vs “1%” of *core* genes is based on debatable calculations—as far as we know, we cannot precisely calculate the amount of LGT (McInerney et al., 2008; Gogarten et al., 2009)–, but serve as the basis to undermine the arguments in favor of the TOL and to highlight the idea of networks. If clades are organismically recognizable, even in microbiology, are we not undermining too much, simplistically, with a very narrow argument? Even if we suppose that most genes were laterally transferred, the genetic-statistical approach is not able to explain why the alleged “1%” genes is so well preserved, neither how this almost negligible percentage would have to be responsible for all the diagnostic traits of major groups, nor why the TOL is consistent under varied data.^5^ We shall insist in a legitimate question: if “99%” of genes are shared, why is diversity identifiable? It may very well be that what has been though as the best biological information, genes and genes-statistics, is too coarse for such questions. If genes are the only information we should center on in this discussion, we should be able to identify all cellular and metabolic differences only with genes-statistics. I think we should be more cautious into these appreciations, have more doubts based on organismal Biology and less certainties provided by gene-centric arguments and by numeric perspectives.

The genetic-statistical approach faces several problems. For example, although the *core* genome of *Escherichia coli* represents only around 50% of its species genome (Rousset et al., 2021) and comprises only around 3000 gene families of the more than 90,000 of its accessory genome (Land et al. 2015 in Blais & Archibald, 2021), it is still an identifiable clade which biologists do not tend to confuse with other bacterial groups. Recognizable groups do not seem to blur because of LGT.^6^  

Networks have been produced by comparing sequences, centered on genetic-statistics. This operation tends to obliterate the relevance of vertical inheritance and its biological consequences—for example, the consistence or cohesion of clades–, as if only genes dispersion counted. However, from an organismal perspective, vertical inheritance is clearly recognizable. Although, gene-centrism assumes that the best representation of genes distribution is the best representation of the history of life, the path of vertical, organismic inheritance, is not well represented in this perspective. We do not see archaean, bacterial or eukaryotic groups blurring against each other, these are clearly distinguishable groups with very specific traits and cellularly distinctive. Neither do we lose diagnostic characters for groups inside the three Domains. Such characteristic features are produced by massive vertical inheritance of genomes. In fact, if LGT was as abundant as vertical inheritance, branching by natural selection would have been impeded. LGT is a strong homogenizing process in evolution, similar in that sense to gene flow. It is important to remind that the TOL results from clusters of vertically inherited genes—the genomic dimension–, from *core* markers (O’Malley & Koonin, 2011). Vertical inheritance, being a predominant hereditary phenomenon that produces branching, cannot be accurately quantified through genes dispersion data alone, as otherwise stated in papers that underestimate it (Doolittle & Bapteste, 2007; Dagan & Martin, 2006; Bapteste et al., 2009; Bapteste & Dupré, 2013; Bapteste & Huneman, 2018). Vertical inheritance is the mechanism by which most of genomes are passed onto the next generation, it cannot be dismissed historically, not even in prokaryotic life, because of LGT being abundant. The TOL is mainly the result of diversification by natural selection with vertical inheritance. LGT and vertical inheritance have two different chronological traces that urge for distinct representations. LGT is always synchronic. In the *horizontally webbed TOL*, both inheritances can be brought together.

## Quantitative and qualitative perspectives and different kinds of inheritance

Considering the arguments presented in subsect. 3.1, the problem about supporting a TOL or networks cannot be well answered by centering the discussion on a quantitative perspective. My position differs from such an approach. If the problem was entirely statistical, and only based on phylogenies made from genomic comparisons, it would seem appropriate to defend networks over the TOL, but the subject is far more complex. There are two recognizable, although related, problems: (1) types of inheritance, and (2) levels of resolution. LGT and vertical inheritance cannot be studied only with quantitative tools, as they respond to different biological processes that produce also distinctive traces, paths,—although they are difficult to identify quantitatively–. I refer here as *trace* to the data that specifically points to vertical or lateral heredity. The trace of vertical heredity is produced by genomic similitudes and core and essential markers shared between related groups, which forms part of the history of a lineage in the TOL. Singular genes spread in different groups becomes the trace that points to LGT. These two types of inheritance require different representations and are recognized by different levels of resolution. The TOL represents clades formed by vertical heredity, reproduction, which transfer entire genomes. The massiveness of gene clusters passed from one generation to the next by this vertical process is retrieved in the TOL from Domains to the smallest taxonomic levels. Such genomic heredity, vertically transferred by reproduction, gives clades a consistency, cohesion, that is identifiable as a particular clade by biologists, may this be a species, a Genus, a Class or even a Domain. If we represent these clades from their shared genes, *heavy* lines result, that is the genomic trace. These are the branches and the TOL’s trunk. Networks cannot produce such a graph. This represents a major resolution level, the level that identifies the enormous number of genes transferred together in vertical heredity, along with its regulation and other biological processes, as metabolism, that produce the internal cohesion in a clade. Along with core genes, this gives us a resolution level that centers on the TOL. Core genes are consistently passed from one generation to the next by genomic transfer in vertical heredity, reproduction. On the other hand, networks depict small sequences transferred from one clade into another, therefore, LGT produces very thin, *light*, lines. LGT lines are thinner than the thinnest branches–species– of the TOL. This is a second and minute resolution level, the genes-specific level. The accumulation of these fine lines produced by LGT over generations might trigger, in many, the idea of wider lines—conflating the two different levels of resolution, as if the information that points to the TOL was the same that what points to networks–. Maybe a metaphor might be useful: this problem is very similar to a tree that, when seen from far, seems entirely white, covered by spider webs—in our case with exclusively horizontal webs—and, if seen closer, the branches appear making the tree distinguishable. Spider webs are not branches, although both coexist intermingled. Recognizing the tree, its structure, its consistency, is one resolution level. Identifying the spider webs is a different resolution level, even if from far away we might only see one thing, the white spider webs. This distinction is useful and necessary, because we are differentiating between the level of genomes, concordant with core genes also, and the level of specific individual genes.

A point must be clarified: when comparing genomes, networks of shared genes arise, but the genomic dimension due to vertical inheritance is what conforms the TOL. This is why searching for *core* genes in genomic comparisons and considering organismal information is important, as it provides the remains of the genomic, vertical, inheritance, although the effects of LGT occurred over long periods of time.

### Why the TOL and networks fail to distinguish between types of inheritance

The exclusively genetic-statistical approach fails to distinguish between the types of inheritance implied. Quantitative data gives us amounts of shared genes between different life groups. But this should not be detached from the biological processes that originate such information. The representation of LGT forming networks cannot be the same as that of vertical inheritance, because of three aspects: (1) LGT is always horizontal, synchronic between individuals living at one singular time, such synchronic event does not have the lineage, generational, dimension; (2) the thickness representing whole genomes and the one that belongs to few genes is far from being similar. Entire genomes transferred thru vertical heredity needs a different representation than that of a few sequences transferred by LGT; (3) vertical inheritance only produces webs in hybridization between different clades. LGT constantly produces webs between branches of the TOL. LGT data, from which we infer networks, is not capable of producing a tree topology. In many representations, like the *noodled web*, all three parameters are misidentified and there is the suggestion that LGT produces an entanglement of branches as *thick*, *heavy*, as those produced by the TOL with its vertical inheritance. There is no biological evidence to support this. Only hybridization lines can be as wide as those of species branches in the TOL. Heavier branches, as Domains or main lineages do not usually collide, although we know of very exceptional cases, such as the endosymbiosis of the mitochondrion or the chloroplast in Eukarya (Margulis, 1992). A *noodled web* suggests that the fusion of different branches is a common event. This would imply that whole genomes from an entire clade pass laterally to another one. Besides the aforementioned examples, inferring that LGT produces fusions of entire genomes—and, even more so, of whole clades– is unfounded.^7^ Some groups of Bacteria show extensive LGT (Cong et al., 2017), but this does not produce the same structure as vertical heredity, nor as branches hybridization. The *noodled web* is an example of underestimating the limits of networks in terms of resolution capacities.

### Why the horizontally webbed TOL can make the distinction between types of inheritance

A depiction that represents both types of inheritance together, distinguishing between different levels of resolution, is possible: the *horizontally webbed TOL*, a TOL with branches interconnected by profuse horizontal LGT thin –light– lines, which is not a network without a TOL, neither just the TOL. LGT does not replace the particular trace of vertical inheritance; both, vertical and lateral inheritances, are indeed there and coexist.^8^ Taking both models, the TOL and the networks, seems like the most accurate representation for the history of life, but not if we lose the conceptual implications of each of them—nor if we obliterate the differences in the data they result from–. Such a representation is not necessarily the most practical for any subject, but in representing historical reconstructions, it is a consistent model with the data we know of and represents more accurately what many other graphs in the area attempt to do. The *horizontally webbed TOL* enables us to distinguish between the different kinds of inheritance, levels of resolution, which leads also to discriminate between whole genomes in clades and small sequences passed from one group on to the next by LGT.

Yet, another problem that demands to differentiate between quantitative and qualitative perspectives is the question of shared genes between biological groups, as well as the sustained presence of some gene groups in the history of life. Some genes are profusely dispersed, representing a big amount of quantitative data, while some others, quantitatively fewer, are sustained in all life. In comparative genomics, recently diverged groups share greater amount of genes, because of vertical inheritance of entire genomes from one generation to the next –*core* genes–. This outlines the trace, or path, of the TOL. In terms of graphics, this is shown as branches. Clades that diverged in earliest periods have less similarities, due to evolutionary processes such as natural selection. The evidence becomes blurrier with chronological distance, in an *information destroying* process, as Elliot Sober described (1988). If we want to retrieve the earliest parts of the TOL—a basic TOL—in spite of LGT, we need, then, to focus on *essential* and on *core* genes that are under high pressures of negative natural selection—NNS– (Estrada et al., 2022). *Essential* and *core* genes are present all along the TOL. The basic TOL, with its major branches and the trunk, results from very specific data, that of *core* and *essential* genes, and points to a distinct information, to organismal clades. *Essential* genes for all life, those genes under the highest pressures of NNS, are useful for characterizing even the LCA (Estrada et al., 2022). Such is the resolution capacity of the TOL: the representation of the divergent evolution produced, principally, by natural selection, with vertical heredity. These results are consilient with what we know about the differences between the three Domains, main cellular distinctions. On the other hand, networks result from tracing genes into groups of genes and genomes comparisons. Many *non*-*essential* genes can be easily received and get fixated by organisms of different clades. Given enough generations, this results in a major phenomenon that entangles the TOL.^9^  

In summary, if we want to have a general model for the history of life, the *horizontally webbed TOL* has the advantage of better representing different levels of resolution—with characteristic weights–, corresponding to different biological processes and data, from vertical and lateral inheritances. Also, it depicts the LGT process as what it is: horizontal.

### Why networks require a TOL to have chronology. The historicity problem

Regarding the discussion on which model better represents life—the TOL or networks–, an important issue arises: historicity, meaning the chronological, contingent changes in lineages, into which biological processes, like natural selection or genetic drift, amongst others, have occurred. The TOL has been always intended to represent the history of life, as a model with an organismal imprint, which is challenging for network reconstructions. The idea that the TOL does not represent the history of life and that networks are, in fact, the most correct historical representations (Dagan & Martin, 2006; Doolittle & Bapteste, 2007) has gained ground in Philosophy of Science (Bapteste et al., 2009; O’Malley, 2010, 2018; O’Malley et al., 2010; Bapteste & Dupré, 2013; Bapteste & Huneman, 2018). These authors claim that networks are the best historical representation, because the TOL would not fully represent the links between different clades:

To be sure, much of evolution has been tree-like and is captured in hierarchical classifications. Although plant speciation is often effected by reticulation (80) and radical primary and secondary symbioses lie at the base of the eukaryotes and several groups within them (81, 82), it would be perverse to claim that Darwin’s TOL hypothesis has been falsified for animals (the taxon to which he primarily addressed himself) or that it is not an appropriate model for many taxa at many levels of analysis. Birds are not bees, and animals are not plants. But in other taxa or at other levels, reticulation may be the relevant historical process, and nets or webs the appropriate way to represent what is a real but more complex fact of nature. (Doolittle & Bapteste, 2007).

In Biology, such a representational change has many implications. If webs are the correct representations and the TOL is not, we do need a new *explanatory toolkit* to decipher and represent life’s history and its inheritance connections, as Ford Doolittle warns us (Doolittle, 2010).

Networks are always the outcome of comparisons of the latest genetic or molecular sequences, a picture of today’s genetic composition of life. We are not visualizing them in the chronological diversification of lineages. Phylogenies are topologies based on similarities from which we infer LGT. Dispersion and similarity, by themselves, are not chronology; we infer chronology from them, but this is done by constructing them over a TOL. We cannot date current-day genes: they may be similar in one group to another’s, but that does not tell us their chronological sequence in and by itself, even if we can infer that a clade, or an operational taxonomic unit (OTU), may be older than another. Consequently, the evidence of webs reflect dispersion, and when trying to infer chronology, we need *core* genes, which belong to genomic vertical inheritance and which produce a TOL. Researchers that have worked on networks, and who have taken them as chronological tracing entities, have systematically represented them as a *noodled web* (Fig. 5, case 1), which already makes it a dubious “not-tree” representation, as discussed previously. As a consequence, this results in a network of life (NOL) that emulates the TOL due to a crucial point: only a tree can give any web chronological polarity and diagonal lines that imply temporality, as only the TOL produces the trace of vertical inheritance. In a network without a TOL, such diagonal lines are unsupported. Networks can be attached to the TOL, which functions as a limiting frame for the diversity of possible webs, as these are produced by LGT, but by organisms that transfer most of their genetic composition by vertical inheritance. Without the TOL, chronology is irretrievable.

Networks have relevant problems regarding historical representations. In the production of networks of life, it has been a common practice to draw diagonal ascending branches—as part of the network and with many of them colliding with other branches—or fine diagonal lines connecting different branches, which are supposed to portray the effects of the LGT (Fig. 5, case 1), *noodled web*, and *diagonally webbed trees* (Fig. 5, cases 2a and 2b). Although in network representations, ascending diagonals might be, of course, intended as didactic, the result is confusing. If, as has been suggested, networks are the correct historical representation, rather than the TOL, and if along with that idea, the LGT data makes it impossible to retrieve a TOL, then it is fair to question from what data does the heavy branches and trunks from the *noodled web* originate? As said before, the colliding branches in this representation are also questionable. The *noodled web* is a relevant representation, it has inspired ideas and has placed the problem of LGT in the center of our discussions, as well as the *diagonal webbed trees* and, of course, they intend to portray an idea, not to be an accurate image, but both have important limitations. The principal one might be, precisely, their misrepresentation of the historical dimension. Data from LGT cannot give a chronological sequence. Both in the *noodled web* and in the *diagonal webbed trees*, LGT represented as diagonal down-up lines, has an unfounded chronology, as if such genes were transferred to the future of other lineages or founded the entire clade. LGT is strictly horizontal, consistent with chronology. Also, in both representations, LGT lines need to be always anchored to a TOL—the chronological structure–. Diagonal lines, common in network representations, aim to replace the TOL with models that are not supported by networks data. Such networks simulate a chronological TOL using data from the TOL but portraying them as made entirely by LGT. This leads to a problematic conceptualization of the history of life. In light of this discussion, it is comprehensible that there is a need for a new *explanatory toolkit* to understand such a question (Doolittle, 2010), but an important part of such a toolkit already exists: it is the TOL supporting the horizontal lines of profuse networks. Some of the attempted solutions, such as networks that emulate a TOL, are misleading. There can be as many layers of horizontal webs as there are generations, although always horizontal, and connected vertically only by vertical inheritance, hence, exclusively represented by the TOL—this is why it matters to differentiate between levels of resolution–. We are certainly interested in giving historical polarity to networks, and we might be able to do so only if we implement them—anchor them—into the TOL. Thus, the historicity of networks relies on a *background TOL* (Estrada et al., 2022). The claim that networks are a better representation of the history of life has, subsequently, two major problems: (1) It mixes one-dimension data with a historical two-dimensions representation, which is systematically inferring a TOL. And (2) Such genetically centered stand privileges a quantitative perspective—genes that appear in an ample amount of data—over the organismic information and a qualitative distinction of *essential* and *core* genes, consistent with organismal data; this distinction is not consilient with networks, but consilient with the TOL. Anchoring the networks into the TOL allows the information from LGT to have a historical dimension. Bringing the TOL and networks together is a very interesting and informative strategy, but clarity in the accompanying narrative is key: such representations are not just a network, but a network assembled over a TOL –a *horizontally webbed TOL–*.

## The *qualitative* TOL

So far, I have defended the *horizontally webbed TOL* as the most conceptually accurate existing representation, as a graphic that allows us to distinguish different levels of resolution: vertical inheritance from the TOL, and LGT from networks. But a problem persists: genetical information is only a part of the phenomenon. How can we best represent the organismal dimension of lineages? Although the genetic-statistics data is central and basic in this endeavor, we need an organismal frame to complement and counterbalance such information, if we want a more precise representation in terms of both, genes and organismic clades. Networks without counterbalances have problems in representing and diagnosing from the level of what taxonomists refer to as *species*, all the way up to the Domains level. For that we must rely on *essential* and *core* genes, which give us a better information about biological groups, and a third aspect: an organismic dimension of the TOL. For this I propose a *qualitative* TOL made from the traits biologists recognize as specific of each major clade and which are not fully represented only through genetics. The organismal perspective of the *qualitative* TOL can counterbalance and enrich the numeric genetic data with information from a different source, which we also use extensively in identifying living forms.

O’Malley and Koonin (2011) proposed the *statistical* TOL, a heuristic tool for recognizing the TOL and frame, with it, the networks. They describe their idea as a fuzzier TOL based on statistical data from sequences similarities. The *statistical* TOL takes multiple genes and compares their similarities and evolution, so that this produces a tree like graph—the idea is represented in Fig. 2, as well as in Fig. 5, case 2a–.^10^ They argue that *essential* markers are not sufficient to sustain a TOL. I agree on a general basis with their idea about the usefulness of the TOL, on the need to frame networks and to have a structure to anchor them, as well as bringing both graphs—the TOL and networks—together. My position might seem similar to theirs, although it differs in a critical point. I defend the relevance of considering organismal data as important as genetic, which is a crucial factor, lacking in the statistical TOL.

For organismal Biology, the LCA would be a cellular ancestor, from which its resulting TOL would comprise lineages of organisms. From a molecular and genetic sequencing approach, and from genomics, the ancestor and the TOL are, obviously, genetic. In a gene-centric perspective, these different entities, organismal and genetic, refer to the same subjects, however the differences between their concepts are critical. Organismic and cellular reconstructions are very appealing, although, again, a differentiation should be made: the genetic LCA and the Last Cellular Common Ancestor—if we can ever find enough evidence for reconstructing the latter hypothesis—are, in fact, different concepts. Actually, the discussion about a single cellular ancestor is extremely challenging, as there are several traits for which we do not know the specific genes involved; however, there is certainly a cellular inheritance produced by cell division (Grimes et al., 1980; Jablonka & Lamb, 2014) and clear cellular differences between the three Domains. Not only does the gene-centrism obliterate the cellular and organismic dimension, but, additionally, the genetic data is much more complicated than what might have been expected. Over the last two decades, the amount of information that contradicts the assumption that genes are enough to understand the history of life has increased, especially as we have come to better understand the relevant effects of LGT (Doolittle 1999a, b; Dagan & Martin 2006; Dagan et al. 2008).

The *qualitative* TOL is coherent with organismic groups, that is its primary characteristic. It derives from the general graph of the *horizontally webbed TOL* but it is made upon genetic and diagnostic organismic information. The concept of the TOL remains a productive research hypothesis, and the qualitative aspects can provide more robustness to it. *Essential* and *core* markers are useful, as well as the organismal dimension, for complementing and correcting the only genetic-statistics information.

Biological Domains constitute a clear example of this problem in the area. From an organismal perspective three are distinguishable, although it has been sustained that genetically we can reduce the distinctions to only two (Watson, 2019; Doolittle, 2020). If genetic data in some representations points to a two Domains TOL, in the *qualitative* TOL diagnostic cellular differences cannot be sub-estimated. Therefore, a two Domains TOL would be an impossible result in a *qualitative* TOL. Clear cell differences between the three major groups of life, Bacteria, Archaea and Eukarya are as central for the *qualitative* TOL as are the genetic data. It is worth mentioning that genetic data is also consistent inside each of the three Domains and between them (as evidenced in several works, for example Woese & Fox, 1977), but when unframed by other information, it leads to unbridled conclusions, loosing resolution refinements. This is why organismic Biology data can constrain genetic information and separate three major clades, rather than concluding one is a subgroup of the other (Eukarya as part of Archaea) because of the similarity of an important group of functional orthologous genes. There are several possibilities for the origin of such genes been shared, which should be treated in a specific study, but paramount cellular singularities in Eukarya with respect to Archaea and to Bacteria should be recognized as importantly as shared genes. The exclusive structural traits of Eukarya, as the nucleus and organelles, Golgi apparatus or endoplasmic reticulum, is overlooked by only comparing sequences, although they constitute important, diagnostic, biological information. The genetic-statistical view would give the idea that all the cellular differences between Archaea and Eukarya are negligible. In addition, this is more questionable when we do not have certainties about the genetic origin of certain cell structures that remain and reproduce by cellular division, and of which we ignore the genetic background. Genetical similarities do not represent well enough clear biological traits. Some important differences are reflected in *essential* and *core* genes, some others are evident at a cellular level. There is a history made by cellular division basic for lineage consistency, that is important to be retrieved and in which the statistical view of genetic markers is not sufficient to account for it, specially because of the *noise* generated by LGT. There are other examples of the above mentioned. With genetic-statistics data we cannot differentiate between metabolisms (Gogarten & Deamer, 2016), nor symbiotic processes (Keeling & McCutcheon, 2017), for example. In contemporary Biology, genes are central to phenotypic, metabolic and regulation traits, but not enough for us to understand those processes. Biological general traits in a population depend not only on a simple gene presence-absence statistics, but on its expression and regulation. Additionally, other related factors, as metabolism and the interaction with the environment, are underestimated or even lost in an only quantitative approach. Analyses that point to a simple gene presence-absence, are unable to indicate which metabolism a specific gene is involved in, as many genes participate in numerous metabolic pathways. These problems are in numerous works and argued upon by some researchers, like the ones Gogarten and Deamer (2016) discuss about on Weiss et al. (2016). Difficulties inherent to such an enterprise are also problematized in works like Becerra et al. (2014). The quantitative TOL cannot differentiate several important organismic histories, for example the products of symbiosis. Mitochondria and chloroplast result as pertaining to Bacteria, which is certainly a part of that history, but researchers have to mend those organismic –not genetic– errors by overruling the genetic data, knowing that these organelles belong to Eukarya. This is, in fact, already attempting a sort of *qualitative* TOL, without making it clear, as the quantitative TOL by itself is unable to give such refined resolution.

The *qualitative* TOL does not conform to the idea that organismic data should have more weight over genetic data, or vice-versa, it calls for complementing one kind of information with the other, to understand where they coincide and to restrain one with the other. If in an only genetic-statistical approach there is the belief that the TOL and the LCA may be reconstructed by using only this kind of data (Estrada et al., 2022), the problem is evident when it results in the incapacity to discern even between existing groups. In reconstructing life history, over-simplifications magnify problems. In reconstructing the LCA, some analyses have given impossible results, because genetic data is not evaluated along with other kind of independent information, as is environmental data (as criticized in Estrada et al., 2022; Gogarten & Deamer, 2016, Goldman & Becerra, 2024). Some of the most common metabolic pathways today are incompatible with the environment in which the LCA lived. Reconstructions need to consider the available ecological information (atmospheric for instance) for those periods, as well as biochemical, metabollic and cellular traits data. This subject has been importantly refined in later years, and has been identified in works that make explicit awareness of such a difficulty (Delaye, 2024 or Carbonell & Peretó, 2025). Not contemplating these problems, centering on genes without these other data frames, may result, as it has, in several unconstrained conclusions. Proposing the LCA had a genome as small as those we only find in pathogens, exemplifies the problem (as pointed out and discussed by Becerra et al., 1997). These problems are retrieved in Estrada et al., (2022), and point to the necessity of contrasting genetic data with organismal information. A next step in the same sense is the *qualitative* TOL. The usefulness of it resides in enriching and guiding, as well as limiting the results provided by genetic-statistical analyses. Even if, in the area, the scientific literature includes statements that assume that only genes provide data for reconstructing the TOL (Koonin, 2003; Weiss et al., 2016; Kim & Caetano-Anollés, 2011) and although the clear importance of genetics and genomics, biological subjects need for less simplistic approaches.

**Fig. 6:**
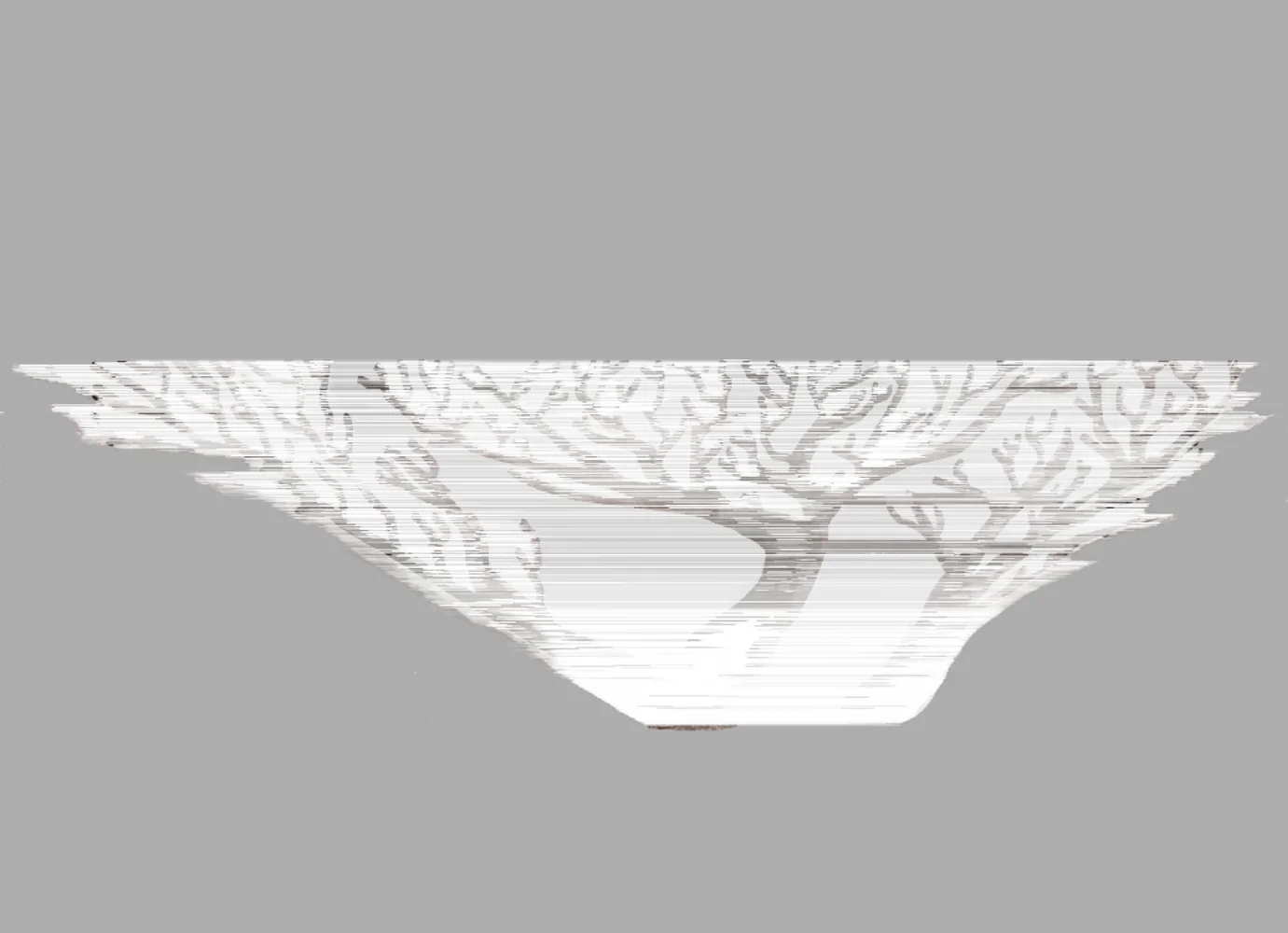
A three domains *qualitative* TOL interconnected profusely by horizontal networks of strong LGT at all levels, then more copiously inside Domains

Assembling organismal data together with genetic-statistical information refines our distinctions of biological lineages, and therefore into more precise phylogenies. The differences between these two models, the *statistical* and the *qualitative* TOLs are not only the two or three Domains representations, but the organismic resolution all along the TOL in the qualitative perspective. In the field, the expression *decorating* phylogenies is used to describe the addition of information to the phylogenetic tree (this is also a problem outlined in Becerra et al., 1997). This is what organismal Biology data does in the *qualitative* TOL, which becomes an interesting tool for *investigative pathways—*in Holmes terms (2004)–, in the reconstruction of life history. Figure 6 shows a representation that I propose for the idea of a *qualitative* TOL.

## Discussion

The differences between an organismal perspective and a gene-centric stand have an impact in the representations of the history of life. Tree representations in Biology are consistent with the pre-molecular era disciplines, data from different areas tend to represent their findings with this basic graphic, from Paleontology to Systematics. Organismal Biology seems to maintain the use of trees as representations, not only in Eukarya, as has often been claimed (Blais & Archibald, 2021 retrieve such discussion), but also in prokaryotic groups. A TOL with three well differentiated cell types—bacterial, archaeal and eukaryotic—remains robust and coherent, as no biologist trained in the field of cellular Biology would confuse Archaea with Eukarya, nor Bacteria with Archaea –after Woese and Fox’s work–. Nonetheless, we shall not forget that the current TOL is not reconstructed under Darwin’s organismal approach, but under a genetic perspective that follows the path of the TOL, albeit with different data—mostly reconstructed from genetic, genomic and molecular information–. As gene-centrism obliterates other sources of data, it is important to remember that genetic reconstructions are unable to accurately account for cellular and organismal evolutionary histories. The belief that genetic markers will provide, by themselves, the history of life, is the historical result of gene-centric biases, which strongly prevails within Biology.

Molecular, genetic, and genomic Biology have provided relevant information about research subjects that were elusive for organismal Biology (Suárez-Díaz 2007 and 2017 center on this subject). Furthermore, phylogenetic studies, based on genetic-statistics, have opened the possibility to work on these two hypotheses—the TOL and the LCA—that could only be approached superficially, mostly as a conceptual proposal, before the development of molecular Biology and genomics.^11^ These two biological disciplines are certainly a key to open this *box* of new subjects, but now we face the problem of attempting to solve the content of such a *box* by using the very same resources, those of a gene-centric Biology. This perspective allows us to evaluate the evolution of specific genes and to compare clades, which tells us about the distribution of genes and the genetic similarities between clades. Although, for inferring ancestry-descent relationships, we also need to retrieve information from organismal Biology. Under a genetic-statistical perspective we might know which genes are the most common today and their distribution—decisive for the study of ancestry—but, without the necessary context, we lack the required information for proposing hypotheses on which genes the LCA had, nor about the history of inheritance of such genes. Many different problems faced by the reconstructions of the LCA are shared with the reconstructions of the TOL, as the LCA is a hypothesis that originates from the TOL.

Some of the aforementioned representations, like 1, 2a, 2b and 4 in Fig. 5, come from works that have defended one idea versus another: they state a disjunctive between networks or the TOL. This constitutes a false dichotomy, since both coexist, are interrelated and, more importantly, refer to different processes. The biological phenomena being portrayed, by the TOL and by networks, are not the same, nor equivalents. This relevant distinction has been systematically conflated in the literature. The TOL and the networks respond to very specific and different types of inheritances that produce contrasting paths; therefore, if we want to better understand the history of life and produce more refined representations, we need a comprehensive graphic that assembles both together, as only networks—reticulations—or only the TOL –bifurcations—cannot depict such diverse histories. The very idea that networks are so abundant that represent better than the TOL the history of life is non concordant with organismal Biology. Such a strong LGT responsible for forming those networks is contradictory with what we know about life diversification and the extensive singularities we find in the different clades. As aforementioned in subsect. 3.1, natural selection has to overcome the homogenizing effect of gene flow to be able to diversify life. Strong LGT—as is believed by many authors to have occurred–, is, in fact, very similar to the effect of gene flow. Our understanding of Biology and evolutionary diversification challenges such idea.

Not only diversification is problematic with such an abundant LGT as is often inferred, but if networks are the correct representation, because of such a profuse LGT, then the evolutionary diversification history cannot be retrieved, as networks cannot indicate us a historical polarity. It seems paradoxical needing to remark that organismal information is central for producing a better understanding of lineages evolution in Biology. Genetic and organismal are very much interrelated but not synonymous.

It must be acknowledged that if we want to portray genes distributions, without a historical interest, networks by themselves are very useful. Also, if our interest is on portraying the main clades in biodiversity, the TOL, without the blurring networks—reticulations–, are helpful, but the subject of this paper is the historical representation.

An important problem emerges from this: the issue is not to decide between networks or the TOL as the best or the right representation, but about what we want to represent. Both are correct and incomplete, as they represent different aspects of life. Some representations and theoretical understandings suggest the merge of both concepts (Swithers & Katz, 2013; Blais & Archibald, 2021), although one of the conclusions in Blais & Archibald, (2021) leads me to reflect upon it:

Though many processes (lateral gene transfer, symbiosis, co–evolution) form patterns incompatible with the tree of life, networks can integrate trees in broader webs of interactions while preserving their topology54. Indeed, trees are a subtype of network, the latter category being more general than, and inclusive of, the former. Opposing trees against networks is thus a *non sequitur*. What really is at stake is whether the network of life is tree–like, reticulate, or a mix of the two. As such, a broader unification of vertical and lateral patterns into a single network is now a tantalizing—if still unrealized—possibility. (Blais & Archibald, 2021)

Two points are worth discussing about the assertion above: (1) I concur that opposing trees against networks is a *non sequitur* and it is also a false dichotomy, because they are constructed with different quality genetic data and imply different processes. (2) Although, indeed, the TOL is a type of network, it has a distinctive trait: it is a partially ordered set—because of chronology–. On the other hand, LGT networks are not chronologically ordered per se, they operate only horizontally, although along many regions of the TOL. So, a tree is a kind of network, but in the specifics, the discussion about TOLs versus networks of life should not be centered only in the reticulation versus tree-like graphs or a mix of both, but on their wider differences rather than only the shape or connections. This involves their meaning and the very difference of the data they originate from. Both are two different graph systems that may coexist within a weighted graphic—as some lines are heavier, thicker than others–, quantitatively. This has also a qualitative implication: different kinds of inheritances—vertical and LGT–, with two different kinds of shapes, in which the TOL has a clear direction, verticality–chronology–. All TOL’s bifurcations occur in the vertical direction, opposed to the networks of life, which produce reticulations in the same level. At any moment of the history of life, the TOL has more weight than the network produced by LGT. Nevertheless, in phylogenies, we see the result of the accumulation of transferred genes through several levels. The massive accumulation of LGT over generations can give the impression that networks and TOL branches have equivalent weights, but this constitutes an observer bias, as well as a conceptual error. The *qualitative* TOL results from thinking on origins and phylogenies, not only on genetic-statistics.

## Concluding remarks

The idea of webs as accurate representations of scientific problems is attractive at this moment in history (García, 2013), and it does seem very appropriate for the results of LGT in phylogenies. However, a different matter is to depict such subjects—despite being difficult and fuzzy—as engulfing and structuring the organismal TOL. The *qualitative* TOL is a tool in which genetic-statistical data, based on *essential* and *core* genes, on sequence similitudes, is complemented with qualitative organismic information and its historical path.

The traditional TOL representation does not account for central genetic and molecular data of LGT. Therefore, networks are much needed. Nevertheless, we must distinguish several major problems: (1) Substitution of the TOL by networks and the supposed historicity of the latter by themselves is unsupported. LGT does not substitute vertical inheritance, neither does it produce the same trace, path: chronology. A solution for this is anchoring the networks onto a *qualitative* TOL as a scaffold, enriched by genetic-statistical data and from organismal Biology, which accounts for cellular differences and organismic lineages. It is important to separate the biological meaning of the TOL and that of networks when addressing historicity, therefore the relevance of the levels of resolution discussion. (2) The *qualitative* TOL, *horizontally webbed* (Fig. 6), is a productive and testable representation, in which vertical heredity—and fusion by endosymbiosis—are the best data for accounting historicity, where LGT processes have to be estimated chronologically by anchoring networks onto the TOL. (3) A TOL that has to be updated and complemented with extensive networks of fine, but abundant, LGT horizontal lines (*horizontally webbed TOL*), seems to be well-supported by evidence. (4) A network that mimics a TOL and which thereby produces a *noodled web* (Figs. 1, and 5, case 1) is in fact using TOL’s evidence. (5) The TOL by itself, without extensive horizontal networks in-between, is beyond rescue as extensive LGT evidence becomes available (McInerney et al., 2008; Gogarten et al., 2009). (6) This TOL and networks problem is a very good example of different processes in need of different representations—levels of resolution, again–, and which, interestingly, can be put together, just as their corresponding biological phenomena. However, this is a relatively young discussion, which can produce some defensive and extreme reactions. (7) The genetic-statistical approach is incapable of discerning such an entangled question. Both positions, defenders of a TOL and defenders of networks have based mainly their arguments on the amount of LGT through the history of life. This is a difficult stand, as LGT has never been accurately measured, but also because, even if LGT is a very abundant process—phylogenies point in that direction–, the only numeric perspective falls short for the explanation of qualitative, organismal processes. The TOL is still a useful concept, as well as are networks, not only because of numeric approximations, but because of biological processes. The genetic-statistics should be one of the exploratory tools, not the explanatory toolkit.

## Data Availability

Not applicable.

## Footnotes

1 It is interesting to retrieve Doolittle’s words on such matter with his critique:

2 In the literature, *Last Universal Common Ancestor* (LUCA) and *Last Common Ancestor* (LCA) are used indistinctly, a point outlined by Becerra et al., 2007. I will use in this paper LCA, as *Last Common Universal Ancestor* is a pleonasm.

3 It is worth mentioning that some authors support at the same time the networks idea with an almost complete loss of the TOL (Dagan & Martin 2006), and extremely detailed LCA reconstructions (Weiss et al. 2016), Martin being the corresponding author of this last work, which reveals a contradiction.

4 As far as we know, there are very few and specific reticulation events that involve TOL branches. Endosymbiosis of mitochondrion and chloroplasts in Eukarya is the collision of two branches of Bacteria with the Eukarya Domain. Also, there are some hybridizations at the level of species, in this case of minor branches.

5 If vertical inheritance is the most massive inheritance, it would also be challenging to separate genes by origins and perform calculations. Knowing which kinds of genes support a TOL and which support networks becomes a relevant research program. The merely genetic-statistical approach makes for a wide bird’s eye view but can only touch the surface of the problem, as it is far from explaining the three Domains stability.

6 A terminology distinction has to be outlined: *essential* genes and *core* genes are used in reconstructions and are not synonymous. *Core* genes refer to those that are shared in genomic comparisons between two or more life groups, they are useful for retrieving the TOL and they can overlap with *essential* genes. On the other hand, *Essential* genes are those that are indispensable for life in a group. The distinction is functional, although in some comparisons *essential* genes conform a part of *core* genes, but this is not true for every comparison.

7 Although, LGT can produce enormous amounts of gene transfers between clades, over numerous generations, I refer here to the amounts of genes in a single event. Colliding branches that form a net of branches, a *noodled web*, imply that the amount of LGT would have been synchronically massive in several, most, branches of the TOL.

8 The problem is similar to what O’Hara (1993) describes as *Systematic Generalization.*

9 On this subject, there is a related idea from other authors, which should be mentioned: the *complexity hypothesis* (Jain et al. 1999, also mentioned in Doolittle & Brunet 2016). This proposal points towards a similar result, concerning *essential* genes, as it states that operational genes are more frequently transferred than informational genes, because the latter “are typically members of large, complex systems, whereas operational genes are not” (Jain et al. 1999). It addresses an empirical outcome: conserved genes seem to be very stable and not easily transferred laterally (Jain et al. 1999; McInerney et al. 2008), such that they have been used for supporting the idea of a TOL:“There are only about 100 genes that are found in all or nearly all prokaryotic genomes and thus have a strong claim to having been present continuously in all lineages [17,18]. These are largely involved with the ribosome and its functions (or transcription), and are considered to be relatively immune to LGT, according to the “complexity hypothesis” [19]” (Doolitle & Brunet 2016).These *core* genes also give cohesion, in the complexity hypothesis, through connecting complex webs of genetical systems.I tend to agree with the c*omplexity hypothesis*, but some clarification might be useful: it is difficult to support the claim that some genes are transferred less than others, as is frequently mentioned. Neither *core* nor *essential* genes are less susceptible to LGT, nor to mutations. *Core* genes are less susceptible to be supplanted and then fixated into other organisms because of strong pressures of NNS, just as we do not account as many mutations in these genes as we find in neutral sequences. Although it may seem obvious, it is worth noting that it is a subject of fixation and depuration, not of transference, nevertheless in the bibliography it is consistently stated as being less transferred, something which would produce many biological doubts with no clear solution. “There are only about 100 genes that are found in all or nearly all prokaryotic genomes and thus have a strong claim to having been present continuously in all lineages [17,18]. These are largely involved with the ribosome and its functions (or transcription), and are considered to be relatively immune to LGT, according to the “complexity hypothesis” [19]” (Doolitle & Brunet 2016). These *core* genes also give cohesion, in the complexity hypothesis, through connecting complex webs of genetical systems. I tend to agree with the c*omplexity hypothesis*, but some clarification might be useful: it is difficult to support the claim that some genes are transferred less than others, as is frequently mentioned. Neither *core* nor *essential* genes are less susceptible to LGT, nor to mutations. *Core* genes are less susceptible to be supplanted and then fixated into other organisms because of strong pressures of NNS, just as we do not account as many mutations in these genes as we find in neutral sequences. Although it may seem obvious, it is worth noting that it is a subject of fixation and depuration, not of transference, nevertheless in the bibliography it is consistently stated as being less transferred, something which would produce many biological doubts with no clear solution.

10 Although the *statistical TOL* has been portrayed as a *diagonally webbed tree*, this is not the outcome of the procedure for producing the *statistical TOL*, but a choice the authors took for their graphic. The *statistical* TOL is able to provide relevant information, especially in terms of different genes that evolve together, which points to the information coming from genomes transferred by vertical inheritance.

11 The reconstruction of ancestral states and the relationship between all the different groups of biodiversity results from the attempt to give a base to a whole life phylogeny, from a TOL.
